# Identification and Capture of Phenolic Compounds from a Rapeseed Meal Protein Isolate Production Process By-Product by Macroporous Resin and Valorization Their Antioxidant Properties

**DOI:** 10.3390/molecules26195853

**Published:** 2021-09-27

**Authors:** Tuong Thi Le, Xavier Framboisier, Arnaud Aymes, Armelle Ropars, Jean-Pol Frippiat, Romain Kapel

**Affiliations:** 1 Laboratoire Réactions et Génie des Procédés, Université de Lorraine, Unité Mixte de Recherche CNRS/Ministère (UMR) 7274, LRGP, F-54500 Vandœuvre-lès-Nancy, France; thi-tuong.le@univ-lorraine.fr (T.T.L.); xavier.framboisier@univ-lorraine.fr (X.F.); arnaud.aymes@univ-lorraine.fr (A.A.); 2 Stress, Immunity, Pathogens Laboratory, SIMPA UR7300, Université de Lorraine, F-54000 Nancy, France; armelle.ropars@univ-lorraine.fr

**Keywords:** macroporous resin, adsorption, rapeseed meal, phenolic compounds, sinapine, sinapic acid

## Abstract

In this study, phenolic compounds from an aqueous protein by-product from rapeseed meal (RSM) were identified by HPLC-DAD and HPLC-ESI-MS, including sinapine, sinapic acid, sinapoyl glucose, and 1,2-di-sinapoyl gentibiose. The main phenolic compound in this by-product was sinapine. We also performed acid hydrolysis to convert sinapine, and sinapic acid derivatives present in the permeate, to sinapic acid. The adsorption of phenolic compounds was investigated using five macroporous resins, including XAD4, XAD7, XAD16, XAD1180, and HP20. Among them, XAD16 showed the highest total phenolic contents adsorption capacities. The adsorption behavior of phenolic compounds was described by pseudo-second-order and Langmuir models. Moreover, thermodynamics tests demonstrated that the adsorption process of phenolic compounds was exothermic and spontaneous. The highest desorption ratio was obtained with 30% (*v*/*v*) and 70% (*v*/*v*) ethanol for sinapine and sinapic acid, respectively, with a desorption ratio of 63.19 ± 0.03% and 94.68 ± 0.013%. DPPH and ABTS tests revealed that the antioxidant activity of the hydrolyzed fraction was higher than the non-hydrolyzed fraction and higher than the one of vitamin C. Antioxidant tests demonstrated that these phenolic compounds could be used as natural antioxidants, which can be applied in the food industry.

## 1. Introduction

Rapeseed (*Brassica napus* L.) is an oilseed cultivated worldwide, particularly in Europe and China (11.684 and 11.760 tons/year, respectively) [1]. This resource shows phenolic compound content as high as 1.705 g of sinapic acid equivalent (SAE)/100 g of dry matter [2]. Naczk et al. found that the proportion of rapeseed polar phenolic compounds was ten times higher than other oilseeds [3]. Rapeseed phenolic compounds are mainly composed of sinapic acid esters. Sinapic acid is a hydroxycinnamic acid widespread in plants [4]. The most abundant rapeseed phenolic compound is an ester of sinapic acid and choline called sinapine [5,6]. Other hydrophilic non-phenolic parts may be associated with sinapic acids such as glucose-based osides (such as sinapoyl glucose) associated or not with kaempferol (such as kaempferol-sinapoyl-trihexoside) [7,8,9]. Rapeseed also contains minor phenolic compounds such as free sinapic acid, ferulic acid, vanillic acid, or syringic acid [3,10]. 

These phenolic compounds are extractible using mild polar solvents such as ethanol, isopropanol, or methanol [9]. A previous study showed that rapeseed phenolic compounds possessed interesting antioxidant effects [11]. However, other authors reported a weak antioxidant effect of sinapine [12,13]. Interestingly, the antioxidant capacity of rapeseed phenolic extracts was reported to be higher after acid or basic treatment [10,14]. These observations suggest that sinapic acid would be far more antioxidant than its esters but there is a lack of comparative data on that point. 

Rapeseed meal is the main by-product of oil industrial extraction processes. Its annual production reached 70 million tons in 2019 (FAO). Rapeseed meal contains around 30% protein, 16% carbohydrates, and 3% of phenolic compounds [15]. The phenolic fraction is composed of polar phenolic compounds, sinapic acid esters (61–70% sinapine, 14–27% SG), and sinapic acid (7–10% SA) [16]. To date, rapeseed meal is used for animal nutrition [17]. Many reports have shown the possibility to use it as a resource for protein isolate production [18]. Protein isolates are protein-based products with a high protein content (>90% on a dry matter basis) used in human nutrition [19]. Rapeseed protein isolate production is classically based on a solid-liquid extraction using aqueous solvents (modulated by pH and ionic strength), a clarification step, and a protein purification step. The purification is most often achieved either by tangential filtration or isoelectric precipitation [20]. Polar phenolic compounds are extracted with proteins during the extraction step and parted from proteins during the protein purification step. At the end of the purification process, phenolic compounds are in an aqueous by-product alongside soluble low molar weight sides, minerals, and non-protein nitrogen-containing molecules. The valorization of this by-product is of crucial importance for the industrial viability of rapeseed protein isolate production. The capture of phenolic compounds could be an interesting valorization strategy. Using salt solution [20] exhibited two purposes: First, this solvent yielded a highly soluble rapeseed protein isolate with high functional properties [20], used in food applications; second, this solution limited the interaction between phenolic compounds presented in the rapeseed meal with proteins. In addition, the salt solution was not toxic to samples and can be easily removed by macroporous resin adsorption.

Macroporous resins are particularly promising for phenolic compounds captured from plant extracts [21,22]. Indeed, it can be easily scaled up and some resins are food grade [23]. The separation with adsorption resins is based on differential affinity between phenolic compounds, impurities, and the adsorbent [24]. The process is also impacted by complex transport phenomena (diffusive and convective transport in the liquid phase, diffusive transport from the liquid phase to the solid adsorbent throughout a limit layer, and diffusive transport inside the adsorbent pores) [25,26]. Some studies have reported the adsorption of rapeseed phenolic compounds on macroporous resins. Most of them used solvent extracts from seeds or meals [8,10]. 

A recent study was focused on rapeseed phenolic capture from an aqueous extract [21]. However, the transport mechanisms were not investigated. Furthermore, there is a lack of comparative data concerning the adsorption of rapeseed phenolic compounds with and without hydrolysis (i.e., esterified sinapic acid and free sinapic acid) and their antioxidant activities.

The aim of this work was therefore to investigate the capture of phenolic compounds from a rapeseed protein isolate by-product obtained by ultrafiltration with or without acidic treatment and to assess their antioxidant activities compared to market references. Phenolic compounds in the aqueous by-product after and before acidic hydrolysis were identified by LC-MS. Five macroporous resins having various properties were screened for phenolic compounds capture. Adsorption kinetics and isotherms were studied in order to elucidate phenolic compounds’ adsorption mechanisms with the more efficient resin. Desorption by ethanol/water mixtures was investigated and obtained purity was considered to choose the most appropriate eluent for phenolic compounds captured from this effluent. Finally, the antioxidant activity of the phenolic fractions obtained after desorption was assessed and compared to sinapine, sinapic acid, and vitamin C standards.

## 2. Results and Discussion

### 2.1. Characterization of the Ultrafiltration (UF) Permeate from Rapeseed Protein Isolate Production and Effect of the Acidic Hydrolysis

#### 2.1.1. Identification of Phenolic Compounds in the Ultrafiltration (UF) Permeate

**Figure 1:**
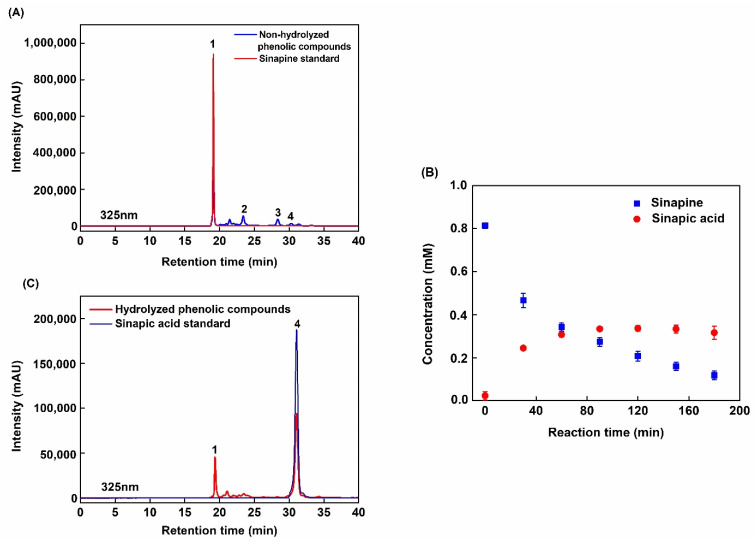
SE-HPLC chromatogram and structures of phenolic compounds detected in the UF permeate obtained from rapeseed protein purification. Chromatograms were recorded at 325 nm. (**A**) Non-hydrolyzed phenolic compounds. (**B**) Kinetic curve of permeate hydrolysis reaction. (**C**) Hydrolyzed phenolic compounds.

**Figure 2:**
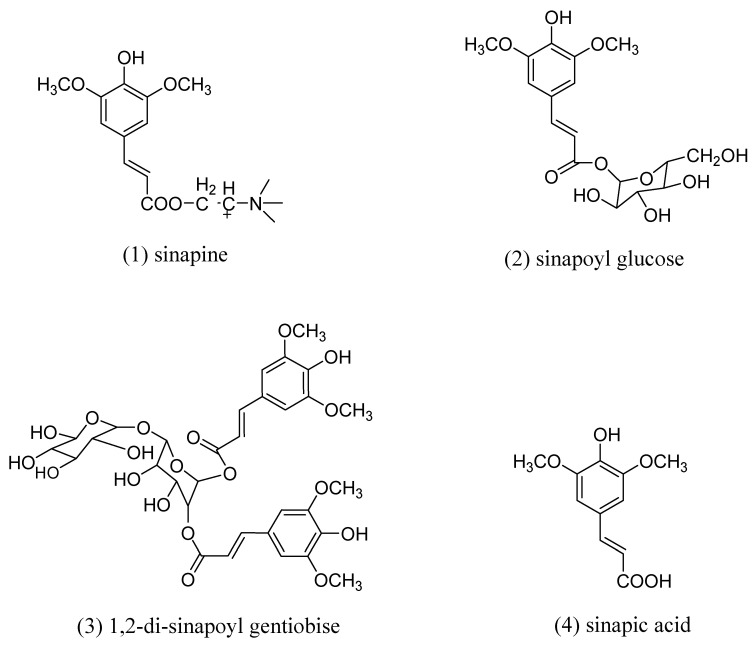
Chemical structures of phenolic compounds (**1**) Sinapine, (**2**) sinapoyl glucose, (**3**) 1,2-disinapoyl gentiobiose, and (**4**) sinapic acid. Different numbers indicated the position of phenolic compounds presented in the HPLC chromatogram.

Figure 1A shows the SE-HPLC chromatogram at 325 nm of the ultrafiltration (UF) permeate. This permeate was obtained from a rapeseed albumin isolate production process recently patented [27,28]. The main peak can be observed at 18.94 min of retention time alongside marginal signals at 23.31, 28.33, and 31.68 min. ESI-MS analysis revealed that this peak contained molecules with *m*/*z* of 310 (positive mode), 385, 753, and 223 (negative mode) (Table 1). This corresponded to sinapine (SP), sinapoyl glucose (SG), 1,2-di-sinapoyl gentiobise, and sinapic acid (SA), respectively. The chemical structures of these phenolic compounds are presented in Figure 2. The identification of sinapine and sinapic acid was confirmed by injection of standards. R. Khattab et al. [8] also identified SP, SA, and SG from canola/rapeseed organic solvent extracts such as 70% ethanol, 70% methanol, or 70% iso-propanol (*v*/*v*). In the UF permeate, SP, other identified SA esters and SA represented 59.78%, 21.66%, and 2.84% in proportion, respectively. Only 15.72% of the phenolic compounds remained unidentified. These observations also agreed with the report from U. Thiyam-Holländer et al. [16]. In their study, the de-oil rapeseed was extracted by distilled water at a ratio of 1:10 (*w*/*v*) for 10 min. The unidentified compounds were most probably other polar sinapic acid esters.

#### 2.1.2. Kinetics of Sinapine Acid Hydrolysis in the UF Permeate 

Figure 1B shows SP and SA kinetics during acidic hydrolysis of the UF permeate. Initial SP concentration was around 0.8 mM (i.e., 0.25 g/L) and continuously decreased during 180 min to reach less than 0.1 mM. SA concentration increased dramatically during the first 60 min and stabilized after 90 min at around 0.35 mM. During this period, the amount of SA increased from 23.35 mg/100 g to 135.18 mg/100 g rapeseed meal. This suggests that the chemical hydrolysis of SP ester bond into SA reached an equilibrium. Hence, the SP concentration decrease after 90 min was surprising. This indicated a degradation into other molecules with no UV properties at 325 nm—this can be hypothesized as a degradation of the benzene ring. Interestingly, at 180 min the purity of SA was the highest (63.66%). SP was still present but at a low proportion (17.44%). Subsequently, this reaction duration was chosen for further study of phenolic compounds capture and bioactivity characterization.

A. Siger et al. [10] found that the contents of sinapic acid after acidic hydrolysis of two rapeseed extracts from two cultivars were 93.2 mg/100 g (cv. Visby) and 89.4 mg/100 g (cv. Bellevue). The SA content observed in our study is slightly higher (135.18 mg/100 g). This could be due to a difference in starting material or to the solvent used for extraction. Indeed, these authors used methanol while we used an aqueous solvent (NaCl 0.11 M at pH 2). This could also be due to a difference in the hydrolysis step duration (during 20 min at 90 °C).

#### 2.1.3. Method Validation

The validation of the SE-HPLC method was performed according to the International Conference on Harmonisation (ICH) of technical requirements for registration of pharmaceuticals for human use [29] recommendations. All experiments were conducted in triplicate. The parameters such as LOD (limit of detection), LOQ (limit of quantification), and standard deviation (SD) values were determined and are summarized in Table 2 using pure standards such as sinapine and sinapic acid.

As listed in Table 2, the LODs of sinapine and sinapic acid were 0.25 µg/mL. The LOQs of sinapine and sinapic acid were 0.6 µg/mL. These low values of LOD and LOQ demonstrated a high sensitivity of the developed SE-HPLC technical method. The linearity of calibration curves was constructed with three measurements for each calibration point ranging from 0.05 to 1.25 mg/mL. A strong linear correlation (R^2^ > 0.99) between the concentration and the peak area was observed. Preliminary results showed that the retention time and peak area were almost similar with intra- and inter-day standard deviation (SD) values less than 0.12 and 1.9 for the retention time and peak area, respectively. These results revealed the good performance of the developed analytical method for quantification of phenolic compounds such as sinapine and sinapic acid within the permeate with and without hydrolysis.

### 2.2. Phenolic Compounds Capture

#### 2.2.1. Resin Screening 

**Figure 3:**
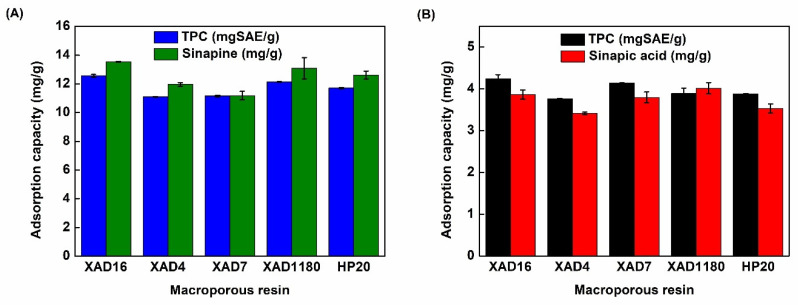
Adsorption capacity of phenolic compounds after adsorption of UF permeate without (**A**) or with acidic hydrolysis (**B**) using XAD4, XAD16, XAD7, XAD1180, and HP20 resins. Results are given in terms of total phenolic compounds (TPC) and the main phenolic compound of the starting product (sinapine and sinapic acid without and with hydrolysis, respectively).

Figure 3 shows the adsorption capacity of total phenolic content (TPC), sinapine, and sinapic acid from permeate and hydrolyzed permeate on XAD4, XAD7, XAD16, XAD1180, and HP20 resins. Table 7 displays the properties of the resins. XAD4, XAD16, XAD1180, and HP20 are nonpolar resins made out of SDVB polymers. They differ by bead and pore size (50–300 Å). XAD7 is composed of acrylamide polymer which is considered a mild polar [26]. All these resins were demonstrated to adsorb phenolic compounds from many different plant extracts [30,31]. The adsorption of TPC from non-hydrolyzed permeate ranged from about 11 (for XAD4 and XAD7) to 12.5 mgSAE/g (for XAD16) of dry resins (Figure 3A). Interestingly, the adsorption of sinapine (main phenolic compound in non-hydrolyzed permeate) was similar to TPC, ranging from 11 (for XAD7) to 13.5 mg/g (for XAD16) of dry resin (Figure 3A). 

Notably, XAD4 showed the lowest adsorption capacity of TPC (about 3.7 mg SAE/g of dry resin) and of sinapic acid (about 3.25 mg/g of dry resin) in hydrolyzed permeate (Figure 3B). On the other hand, XAD16 showed the highest adsorption capacity of TPC (4.1 mgSAE/g of dry resin) and of sinapic acid (3.9 mg/g of dry resin) (Figure 3B). 

The adsorption capacity depends on a molecule’s affinity toward the material and the specific surface [32]. This last parameter is conditioned by bead size and can be modulated by pore size if it limits the molecule’s inner accessibility [26]. Pore ≤ 50 Å was shown to limit the diffusion of phenolic compounds into particle pores by steric hindrance [29,32]. Among styrene-divinyl benzene (SDVB) resins, XAD16 showed the highest specific area (900 m^2^/g) followed by XAD4, XAD1180, and HP20. Interestingly, this ranking corresponded to observed capacity values. This indicates that SDVB resin capacities were essentially governed by the specific area. It can be hypothesized that XAD4 low pore size (50 Å) did not yield additional diffusion limitations. The adsorption behavior of phenolic compounds in non-hydrolyzed and hydrolyzed permeates onto SDVB resins and mild polar resin (XAD7) had the same pattern. Probably, the interaction of sinapine (Figure 2) (a main phenolic compound in non-hydrolyzed permeate) and sinapic acid (a main phenolic compound in hydrolyzed permeate) (Figure 2) and SDVB resins were through pi–pi stacking interaction (aromatic ring part of phenolic compounds and benzene rings of SDVB resins) [26]. XAD16 and XAD1180 showed the highest adsorption capacity of TPC from non-hydrolyzed and hydrolyzed permeates. However, XAD16 had the highest adsorption capacity of sinapine and sinapic acid. The very high specific area of XAD16 (900 m^2^/g) showed better adsorption in terms of massic capacity than XAD7 or HP20 with a similar specific area (450 or 500 m^2^/g). So, the adsorption kinetics, isotherms, thermodynamics properties, and phenolic compounds desorption were further investigated with this resin.

#### 2.2.2. Adsorption Kinetics 

**Figure 4:**
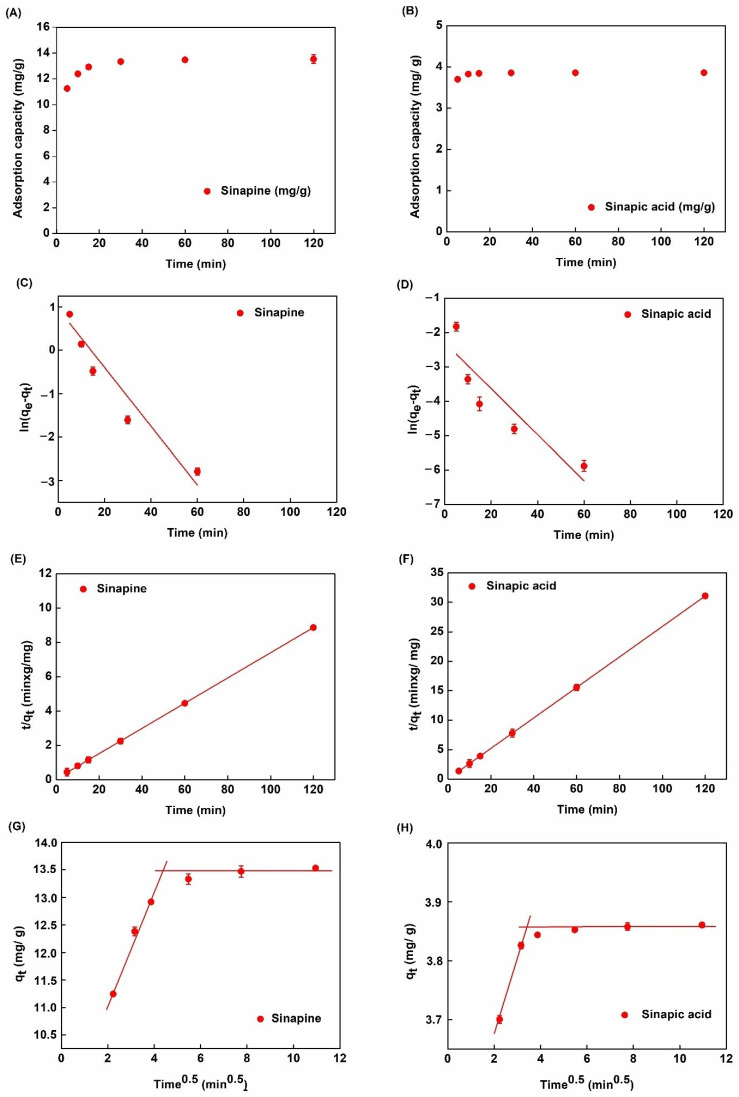
Adsorption kinetics with XAD16. (**A**,**B**) Adsorption kinetic curve. (**C**,**D**) Pseudo-first-order model. (**E**,**F**) Pseudo-second-order model. (**G**,**H**) Intra-particle diffusion model (in linearized forms) of SP and SA in non-hydrolyzed and hydrolyzed permeates, respectively.

Adsorption kinetics of the main phenolic compounds from UF permeate with (sinapic acid) or without hydrolysis (sinapine) on XAD16 are presented in Figure 4A,B. Very similar trends were observed. A large part of phenolic compounds was quickly adsorbed (up to 90% of the equilibrium capacity in 30 min). Then, the kinetics slowed down up to the equilibrium observed between 60 min and 120 min. As expected from the resin screening results, this indicated that XAD16 has a strong affinity toward rapeseed phenolic compounds. Such behavior was also reported for adlay bran phenolic compounds [30].

Adsorption kinetics were regressed with pseudo-first-order (PFO, [33]) and pseudo-second-order (PSO, [34]) equations in linearized form (Figure 4C–F). Table 3 summarizes the corresponding equations, model parameter values, and R^2^ of the linear regressions. 

The R^2^ obtained with linearized PFO (ln(*q_e_*−*q_t_*) vs. *t*) was less than 0.94 for both sinapine and sinapic acid. Furthermore, the calculated *q_e_* for XAD16 (0.32 mg/g and 0.29 mg/g for sinapine and sinapic acid, respectively) was found to be very different from experimental values (13.52 and 3.86 mg/g for sinapine and sinapic acid, respectively). On the other hand, the R^2^ of the linear regression of the *t/q_t_* vs. *t* plot was very close or equal to 1 (0.999 and 1). Moreover, the calculated *q_e_* (13.54 and 3.86 mg/g for sinapine and sinapic acid) predicted from the PSO model was very near the experimental values of *q_e_* (13.53 and 3.86 mg/g for sinapine and sinapic acid). These results indicate that adsorption kinetics followed a PSO model for the XAD16 resin. This was also observed with adsorption of chlorogenic acid from by-product protein isolates on XAD16 resin [26].

Solute transport phenomena are complex in adsorption processes. In the liquid phase, solutes are transported by convection and diffusion. There is also diffusive transport from the liquid phase to the bead surface (through a limit liquid film) and diffusive transport inside the particle’s pores. Adsorption kinetics may be modulated by several diffusional types of transports. The intra-particle diffusion model [35] is commonly used to investigate the diffusive rate-controlling phenomenon [25,36].

Figure 4G,H show *q_t_* vs. *t*^0.5^ plots obtained with the XAD16 resin. These plots correspond to the linear form of the intra-particle diffusion model (Table 2). The slopes represent the constant rate (*k_i_*) of each adsorption step while *C_i_* (intercept at *y*-axis) is related to the thickness of the limiting layer. R^2^, *k_i_*, and *C_i_* values obtained from linear regressions are displayed in Table 3. Interestingly, *k*_1_ (0.103 and 0.09 (mg/g)/min^0.5^) for sinapine and sinapic acid, respectively) are by far higher than *k*_2_ (approximately 0). These differences might come from the smaller molecular size of sinapic acid than sinapine. It can also be noticed that R^2^ values for sinapine and sinapic acid are 0.9828 and 0.9639, respectively. This indicates that for both liquid effluents, the adsorption process is limited by two diffusional effects. In the previous study, a similar pattern was also observed with the adsorption of chlorogenic acid onto XAD16 resin [26]. Very similar results were observed with the adsorption of alfalfa phenolic compounds on HP20 and AER1 resins [25]. It was interpreted as a two steps adsorption process. The first one is related to the diffusional transport throughout the boundary layer at the liquid/bead interface. Its high rate constant (*K*_*i*,1_ was 1.03 and 0.09 (mg/g)/min^0.5^) for sinapine and sinapic acid, respectively) indicates a low diffusional limitation at this stage. The second one is due to intraparticle diffusion. The low rate constant (*K*_*i*,2_ approximately equal to 0 for both liquid effluents) indicates a stronger diffusional limitation. Such observations and explanations were also made by others [31,37].

In this study, our results revealed that the adsorption capacity could reach the equilibrium state after 30 min of adsorption time. The adsorption of phenolic compounds such as sinapine and sinapic acid was followed by a pseudo-second-order model. As discussed in our previous study [26], the adsorption of these phenolic compounds is based on the pi–pi stacking interaction between the benzene ring of phenolic compounds and benzene rings macroporous resin (XAD16). Therefore, this adsorption was considered as spontaneous and physical adsorption [26].

### 2.3. Adsorption Isotherms 

**Figure 5:**
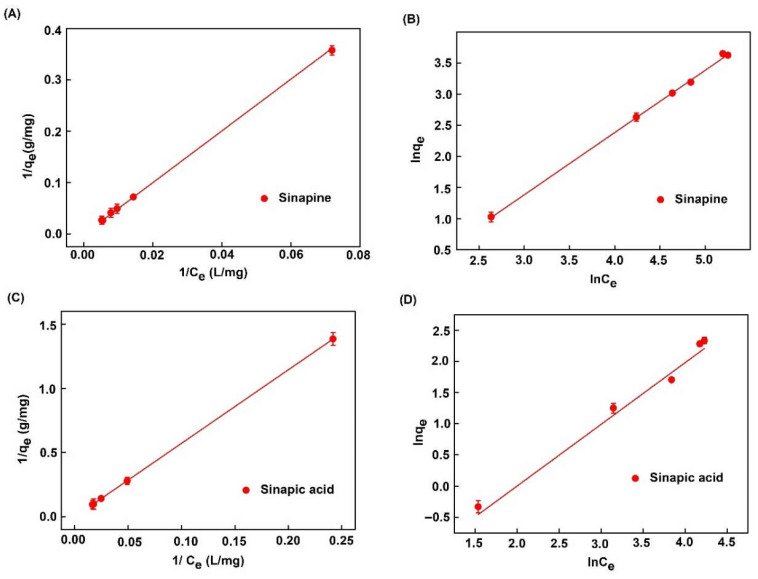
Adsorption isotherms of phenolic compounds on XAD16 with Langmuir and Freundlich linear models. (**A**,**B**) Langmuir and Freundlich models for sinapine. (**C**,**D**) Langmuir and Freundlich models for sinapic acid.

Figure 5 shows adsorption isotherms of sinapine and sinapic acid onto XAD16 at 25 °C. Data were regressed with Langmuir (Figure 5A,C) and Freundlich (Figure 5B,D) equations as previously carried out elsewhere [24,38,39]. Table 4 lists the R^2^ of the regression, equations, and model parameters with sinapine and sinapic acid. R^2^ values indicated that experimental data were better fitted by the Langmuir model (0.997 and 0.9999 for sinapine and sinapic acid, respectively) than by the Freundlich model (0.996 and 0.9978 for sinapine and sinapic acid, respectively). This indicated that the same adsorption mechanism took place in any case. This consisted of monolayer adsorption of phenolic compounds at the surface of the resin [24,38,39]. These findings are also in agreement with another study on chlorogenic acid adsorption on XAD16 resin from sunflower meal [26].

Moreover, this finding is consistent with that of M. Moreno-González et al. [21] regarding the adsorption of sinapic acid from canola/rapeseed meal using the FPX66 resin. The maximum adsorption capacity of sinapine and sinapic acid was 35.93 mg/g (0.12 mol/g) and 23.96 mg/g (0.11 mol/g) of dry resin, respectively (Table 4). The values obtained in this study suggest that the adsorption onto resin material implies interaction with the same part of the molecule (sinapine and sinapic acid) without the interference of the choline part (quaternary ammonium group, Figure 2). This observation has never been published. Meanwhile, according to M. Moreno-González et al. [21], the maximum adsorption capacity for sinapic acid was lower (about 15 mg/g or 0.07 mol/g) onto the non-polar (SDVB) FPX66 resin. The different values might be due to other organic compounds such as carbohydrates, amino acids, and proteins in raw materials. It might also be due to the impact of ionic strength. Moreover, the difference in physical characteristics also caused the difference in adsorption capacity. A. Thiel et al. [40] investigated the adsorption of sinapic acid and other compounds onto zeolites and hydrophobic resins, including XAD16, the resin used in this study. These authors claimed that the adsorption capacity of sinapic acid was higher than the one presented here (44.5 mg/g or 0.20 mol/g of dry resin). However, these authors evaluated the experiments at different pH (pH 5) and used different starting materials; rapeseed meal extracted with heated deionized water at 70 °C at a ratio of 1:8 (solid:liquid) that might explain the difference in these values.

### 2.4. Determinations of Thermodynamic Parameters

**Figure 6:**
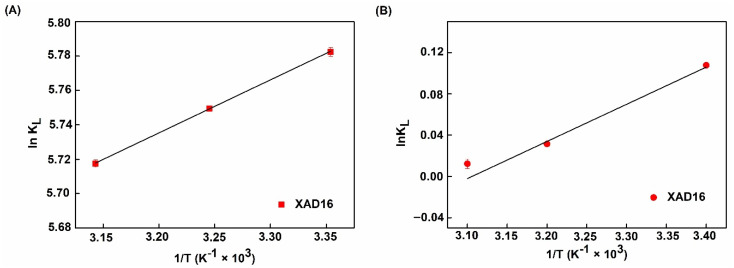
ln *K_L_* vs. 1/T plot of adsorption equilibrium constant *K_L_* using Langmuir isotherm of (**A**) Sinapine and (**B**) Sinapic acid.

The effect of temperature on the adsorption capacity of phenolic compounds from the XAD16 resin in the two liquid effluents was investigated to obtain the thermodynamic parameters of adsorption. Langmuir model parameters and R^2^ are listed in Table 5; ∆H and ∆S were determined through the slope and intercept of ln *K_L_* against 1/T (Equations (2) and (3)) (Figure 6A,B) according to Van Hoff’s equation. Enthalpy changes (∆H) for the sinapine and sinapic acid adsorption process on XAD16 resin were −2.56, and −2.72 kJ/mol, respectively (Table 5). Negative values indicate an exothermic adsorption process. The fact that values were less than 43 kJ/mol demonstrates that the adsorption process of phenolic compounds on the XAD16 resin was governed by physical rather than chemical interactions [30]. This demonstrates that the XAD16 resin would not undergo structural changes during the phenolic compounds’ adsorption process. Therefore, the adsorption of phenolic compounds on the resin only takes place through a physical mechanism with no chemical reactions. This observation was also reported by Z. P. Gao et al. [41] who studied the adsorption of polyphenols from kiwifruit juice using AB-8 resin [41]. In addition, the entropy changes (∆S) values of XAD16 were −55.95 and −8.37 kJ/molK for sinapine and sinapic acid, respectively. These negative values suggest a random adsorption process at the solid–liquid interface [41] which occurred due to the desorption process of water molecules previously adsorbed onto the resins’ surface [41]. The negative free energy change (∆G) deduced from ∆H and ∆S (Table 5) suggests that phenolic compounds’ adsorption onto the XAD16 resin was a spontaneous process. Moreover, the absolute value of ∆G < 20 kJ/mol confirmed the physical adsorption of phenolic compounds onto XAD16 resin [41,42].

### 2.5. Desorption of Phenolic Compounds from the XAD16 Resin

**Figure 7:**
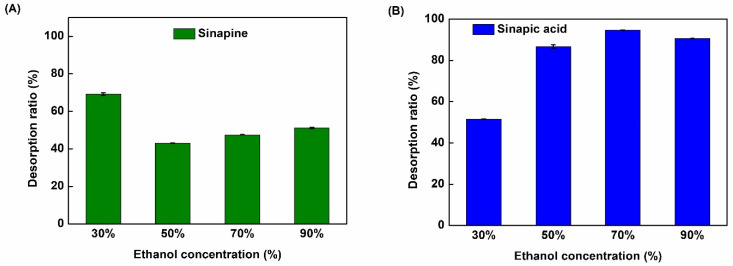
Desorption ratio of sinapine (**A**) and sinapic acid (**B**) from the XAD16 resin at different ethanol concentrations.

To assess the effect of ethanol concentration on desorption, five ethanol concentrations were evaluated: 30, 50, 70, and 90% (*v*/*v*). Preliminary results showed that the desorption curve reaches equilibrium after 120 min (data not shown). As shown in Figure 7, ethanol concentration significantly influenced SP and SA desorption ratio. The highest desorption ratio of sinapine and sinapic acid was observed with ethanol (30% and 70% (*v*/*v*) (*p* < 0.05), respectively). These results indicate that the desorption ratio was influenced by the ethanol concentration and the solubility of phenolic compounds in the desorption phase. Indeed, sinapine is a polar compound demonstrating a good desorption ratio with ethanol at a lower concentration. Sinapic acid is by far less polar, therefore, presenting a higher desorption ratio at a higher ethanol concentration. Surprisingly, the desorption ratios of sinapic acid and sinapine in this study were much higher than in the report of A. Thiel et al. [14,40] (about 44% and 3.7% for SA and SP, respectively) who used the same macroporous resin (XAD16) and desorption with 70% aqueous ethanol. However, a high desorption ratio of sinapic acid was also found by M. Moreno-González et al. [21] with 70% (*v*/*v*) of ethanol.

**Figure 8:**
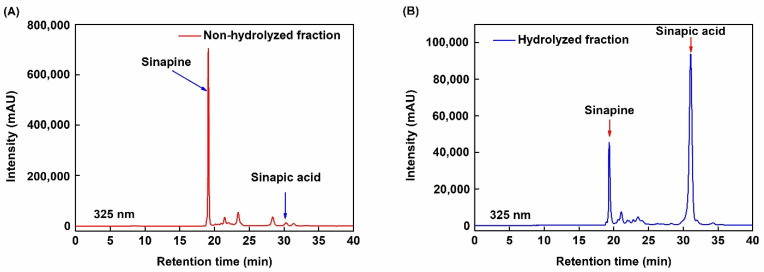
HPLC chromatogram of phenolic compounds in (**A**) the non-hydrolyzed fraction after desorption with ethanol 30% (*v*/*v*) and (**B**) the hydrolyzed fraction after desorption with ethanol 70% (*v*/*v*).

Figure 8 shows the HPLC chromatogram of phenolic compounds after purification with the XAD16 resin. The peaks of sinapine (Figure 8A) and sinapic acid (Figure 8B) are highlighted in the desorption fractions. Sinapine (63.31%) and sinapic acid (73.00%) are the mains phenolic compounds in the hydrolyzed fraction after purification. As different compositions of phenolic compounds after purification might lead to different biological actions, we used both fractions to assess the antioxidant activity in subsequent experiments.

### 2.6. In Vitro Antioxidant Activity

**Figure 9:**
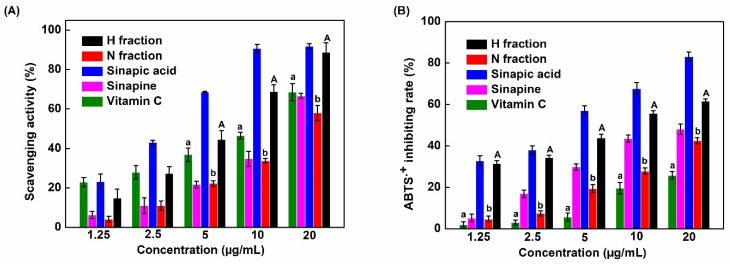
Scavenging activity of the non-hydrolyzed (N fraction) and hydrolyzed (H fraction) fractions compared to pure sinapine, sinapic acid, and vitamin C determined using (**A**) DPPH assay and (**B**) ABTS assay. Bars labeled with the different lowercase letters and uppercase letters are significantly different (*p* < 0.05).

The free radical scavenging rate of DPPH was determined to assess the antioxidant activity of the non-hydrolyzed and hydrolyzed fractions by comparison to pure SP, SA, and vitamin C. As shown in Figure 9A, the scavenging rate increased with the concentration of non-hydrolyzed fraction (N fraction). The same was observed for the hydrolyzed fraction (H fraction), but in this case, interestingly, we observed scavenging rates higher than the ones obtained with vitamin C at 5, 10, and 20 µg/mL. These results were confirmed by the ABTS assay (Figure 9B).

Table 6 presents IC50 values of compounds tested using DPPH and ABTS assays. These data demonstrate that the antioxidant activity of the hydrolyzed phenolic fraction was stronger than the one of the non-hydrolyzed fraction and vitamin C in both assays. These findings are likely due to the enrichment of the hydrolyzed fraction in sinapic acid as this compound exhibited the lowest IC50 values.

It has already been shown that phenolic compounds found in RSM are excellent antioxidants. Previous studies showed that the antioxidant activity is most likely due to vinylsyringol found in rapeseed oil [16,43]. In this study, we show that sinapic acid also has strong antioxidant properties. Interestingly, the hydrolyzed fraction (containing mainly SA) was more effective than the non-hydrolyzed fraction (mainly containing SP) and vitamin C. These observations are in accordance with the report of S. Vourela et al. [44] who investigated the antioxidant function of phenolic compounds in rapeseed oil. Other researchers [10,12,45] and our previous study [46] also indicated that the high concentration of phenolic compounds is related to a high antioxidant capacity. Taken together, these data indicate that phenolic compounds isolated from RSM are interesting natural antioxidants that could be used in the food industry or other applications [47].

## 3. Materials and Methods

### 3.1. Materials

The rapeseed meal was provided by Olead (Pessac, Bordeaux, France). Sinapine standard was purchased from ChemScience (JJ08852, Monmouth Junction, NJ, USA). Sinapic acid and formic acid (FA) were obtained from Sigma-Aldrich (St. Louis, MO, USA). Sodium chloride (NaCl) and sodium hydroxide (NaOH) pellets were purchased from VWR (Radnor, PA, USA). Chlorohydric acid (HCl) 37% was from CarloErba (Val-de-Reuil, France). Acetonitrile (ACN) solution was provided by Biosolve BV (Valkensward, Netherlands). Absolute ethanol (EtOH) was purchased from DASIT (Paris, France). All solvents and chemical reagents used in this study were analytical and high-performance liquid chromatography (HPLC)-grade. 1,1-diphenyl-2-picrylhydrazyl (DPPH), 2,2′-azino-bis-(3-ethylbenzothiazoline-6-sulfonic acid) (ABTS), potassium persulfate, and ascorbic acid (vitamin C) were provided by Sigma-Aldrich (St. Louis, MO, USA).

Five macroporous resins (XAD7, XAD4, XAD16, XAD1180, and HP20) were purchased from Sigma-Aldrich (St. Louis, MO, USA). The characteristics of these macroporous resins are shown in Table 7.

### 3.2. Rapeseed Protein Isolate and Its Aqueous By-Product Production

The protein isolate/purification process was performed in two steps: First, protein extraction from rapeseed meal (RSM), and second, protein purification by ultrafiltration as recommended in [27,28]. For protein extraction, the appropriate amount of RSM and NaCl 0.1 M solution was mixed at a solid/liquid ratio of 1:9 (*w*/*w*). The extraction step was performed at pH 2 under agitation at 400 rpm for 30 min. Then, the liquid extract was clarified by centrifugation at 10,000 *g* for 30 min at 20 °C (Heraeus Megafuge 16R centrifugation system, ThermoScientific, Waltham, MA, USA). The supernatant was further clarified by tangential microfiltration using a 0.22 µm membrane at 0.2 bar of transmembrane pressure (0.1 m^2^ area, Pellicon Mini Cassette Durapore, polyvinylidene difluoride (PVDF) membrane, MA, USA). The protein purification was achieved by diafiltration using an Äkta Flux 6 device (GE Healthcare, Chicago, IL, USA) at a feed flow of 2 L/min and a transmembrane pressure of 1.5 bars. During the process, the retentate compartment was fed with 0.5 M salt solution at the same flow rate as the permeate flux in order to keep the volume constant. Six diavolume (DV, DV = V_NaCl_/V_o_) of NaCl solution was poured. Then, three DV of deionized water was used to flush NaCl from proteins. The permeate containing the phenolic compounds was recovered and adjusted to pH 2 by adding HCl 1M for further studies.

### 3.3. Phenolic Compounds Hydrolysis

Phenolic compounds contained in the UF permeate were hydrolyzed under acidic conditions as suggested by A. Siger et al. [10]. To do so, 350 mL of permeate was mixed with 70 mL of HCl 37% (*v*/*v*). The reaction was carried out at 75 °C with agitation at 150 rpm for 3 h. During the reaction, 600 µL of samples were taken at 30, 60, 90, 120, 150, and 180 min for phenolic compounds analysis and quantification by SE-HPLC. 

### 3.4. Analytical Methods

#### 3.4.1. Phenolic Compounds Identification by HPLC-MS

Phenolic compounds from UF permeate with or without acidic hydrolysis were separated by SE-HPLC and identified by ESI-MS according to T. T. Le et al. [26,46]. The HPLC system used was from Shimadzu Corporation (Kyoto, Japan), equipped with a pump and degasser (LC-20AD), a column oven (CTO-20A), and a diode array detector (CPO-M20A). The analysis was performed using a size exclusion (SE) column Biosep 5 µm SEC-s2000 145 Å column (300 × 7.5 mm, 5 µm) purchased from Phenomenex (Torrance, CA, USA). The elution was performed for 40 min at 35 °C using ACN:UltraPure water:formic acid with a 10:90:0.1 (*v*/*v*) composition. The flow rate was 0.6 mL/min. The injection volume was 5 µL. ESI-MS analysis was performed online with an ion trap (IT) mass spectrometer apparatus LC-MS2020 (Shimadzu Corporation, Kyoto, Japan). The nitrogen flow rate was set at 21.5 L/min. Data were analyzed using the LabSolution package. Qualitative identification of phenolic compounds from permeate and hydrolyzed permeate was based on *m*/*z* values and unique fragmentation pattern of the protonated molecules ions [M − H]^+^ or [M − H].

#### 3.4.2. Sinapic Acid (SA) and Sinapine (SP) Quantification by SE-HPLC

SA and SP were quantified from UF permeate with or without acidic hydrolysis by the above described SE-HPLC method using their UV signal at 325 nm. To do so, calibration curves were established by quantifying the peak area of standard SA and SP at concentrations ranging from 0.05 to 1.25 mg/mL. The linear regression equations of the calibration curves were y = 2.9.10^7^x and y = 5.31.10^7^x with R^2^ = 0.9998 and R^2^ = 0.9984 of SP and SA, respectively.

#### 3.4.3. Method Validation

Validation of this method was conducted according to the International Conference on Harmonization (ICH) [29]. Linearity of the detector responses and limits were performed in the range 0.05–1.25 mg/mL. Each calibration plot performs an average of three independent repetitions for five different concentration levels. Linear regression analysis was used to determine the slope and correlation coefficient (R^2^). All experiments were tested three times for phenolic compounds standards and the phenolic compounds presenting in samples solution in this study. 

#### 3.4.4. Total Phenolic Contents (TPC)

The quantification of total phenolic content (TPC) was performed by the “sum of phenolic acids” method of R. Khattab et al. [8]. TPC was estimated as SA equivalent (SAE) from the sum area of all peaks of phenolic compounds at 325 nm using the above described SE-HPLC method.

#### 3.4.5. NaCl Content

NaCl content in UF permeate was evaluated by measuring each sample’s conductivity using a conduct meter (MeterLab HPM210, Radiometer analytical, Lyon, France). NaCl solutions with concentrations ranging from 0.2 to 50 g/L were used to establish the calibration curve. The calibration equation was y = 1.4088x and the coefficient of determination was 0.993.

#### 3.4.6. Total Carbohydrate Content

Total carbohydrate content in UF permeate and hydrolyzed permeate was determined according to the anthrone-sulfuric acid method of E. W. Yemm and A. J. Wilis [48]. Glucose was used as standard and glucose solutions with concentrations ranging from 0.1 to 1 mg/mL were used to construct the calibration curve (y = 1.1845x, R^2^ = 0.9981).

#### 3.4.7. Protein Content

Protein content in UF permeate was assessed using the Kjeldahl method (AOAC, 1995) [49]. These compounds were low molar weight peptides or free amino acids since proteins were fully retained by the membrane. A nitrogen-to-protein factor of 6.25 was used as frequently used for rapeseed meal.

### 3.5. Adsorption/Desorption Study

#### 3.5.1. Resin Screenings

The resins were screened on the basis of massic adsorption capacity (*q_e_*, amount of phenolic compounds adsorbed per g of resin) calculated as:

(1) $$q_{e}=\frac{\left(C_{0}-C_{e}\right)V_{i}}{W}$$

where *C*_0_ and *C_e_* are the initial and equilibrium concentrations of phenolic compounds in non-hydrolyzed and hydrolyzed permeate solution, respectively (mg/mL); *V_i_* is the initial volume of permeate added onto the resins (mL); *W* is the weight of the dried resin (g).

#### 3.5.2. Adsorption Kinetics

The adsorption capacity was monitored after 5, 10, 15, 30, 60, 90, and 120 min. To do so, phenolic compounds’ concentration was measured in the liquid phase by HPLC. *q_e_* was deduced from the concentration. Results were plotted under linearized models (pseudo-first-order, pseudo-second-order, and intra-particle diffusion).

#### 3.5.3. Adsorption Isotherms

Adsorption isotherms expressed the relationship between phenolic compounds’ adsorption capacity (*q_e_*, mg/g) and the concentration of sample solution in the liquid phase at the equilibrium (*C_e_*, mg/L). For the adsorption study, a duration of 120 min was chosen. Experiments were carried out at 25 °C. Langmuir and Freundlich models were used to regress experimental data.

#### 3.5.4. Adsorption Thermodynamic Parameters

The effect of the temperature was investigated by determining the adsorption isotherms at 298.15, 308.15, and 318.15 K. Enthalpy and entropy variations were obtained from the slope and intercept of the linear plot lnK_eq_ vs. 1/T according to the linear form of Clausius–Clapeyron Equation (2):

lnK_eq_ = −∆H/RT + ∆S/R (2)

where lnK_eq_ is the natural logarithm of the constant of adsorption equilibrium (K_eq_), ΔH is the enthalpy change (J/ mol), ΔS is entropy change (J/mol), R is the universal gas constant (8.3144 J/molK), and T is the absolute temperature in Kelvin (K). 

∆G was determined using Equation (3):

∆G = −RT lnK_eq_ (3)

where ΔG (J/mol) is the Gibbs energy change.

#### 3.5.5. Desorption

Desorption ratios were determined using different water/ethanol solutions after the adsorption step on XAD16 up to equilibrium. Forty milliliters of 30, 50, 70, and 90% water/ethanol (*v*/*v*) was added to the resin and shaken at 150 rpm and 25 °C for 2 hours to reach desorption equilibrium. Resins were washed twice with deionized water prior to solvent addition. Resins were separated from the liquid filtration using filter paper. Phenolic compounds concentration in the liquid was quantified by HPLC. The desorption ratio was calculated using Equation (4):

(4) $$Desorption\;ratio\;\left(\%\right)=\frac{C_{d}V_{d}}{\left(C_{0}-C_{e}\right)V_{i}}100\%\;$$

where *C_d_* is the concentration of CGA in desorption solution (mg/mL) and *V_d_* is the volume of the desorption solution (mL).

### 3.6. Antioxidant Activity

#### 3.6.1. DPPH Radical Scavenging Assay

DPPH free radical scavenging activity was carried on according to C. Wu et al. [50] with some modifications. Briefly, DPPH 0.2 mM in MeOH was prepared. one hundred microliters of samples at 1.25, 2.5, 5, 10, and 20 µg/mL was mixed with 100 µL of DPPH 0.2 mM and added to the wells of a microplate. The mixtures were kept at 25 °C for 30 min in the dark and then shaken for 30 s. The absorbance was measured at 517 nm. The inhibition percentage (%) of radical scavenging capacity was expressed as follows:

(5) $$DPPH\;radical\;scavenging\left(\%\right)=\frac{\left(A_{DPPH}\;-\;A_{blank}\right)-\left(A_{sample+DPPH}-A_{sample+blank}\right)}{A_{DPPH}-A_{Blank}}100\left(\%\right)$$

where *A_DPPH_* is the absorbance of the DPPH solution, *A_blank_* is the absorbance of pure methanol, *A_sample+DPPH_* is the absorbance of DPPH with the sample, and *A_sample+blank_* is the absorbance of pure methanol with the sample.

#### 3.6.2. ABTS Radical Scavenging Assay

The ABTS assay was used according to the procedure described by R. Re et al. [51] with some modifications. ABTS solution (3.5 mM) and potassium persulfate (1.225 mM) were mixed to produce the ABTS^+^ solution. This solution was kept at room temperature in the dark for 16 h before use. When the radical had formed, the absorbance of ABTS^+^ solution at 734 nm was adjusted to 0.7 ± 0.02 by dilution with 95% (*v*/*v*) methanol solution with a 1:32 ratio (*v*/*v*). Twenty microliters of samples at different concentrations (20, 10, 5, 2.5, and 1.25 µg/mL) was mixed with 180 µL ABTS^+^ solution and added into each well of a 96-well plate. After 5 min of incubation in the dark at 25 °C, the plate was shaken for 20 seconds and the absorbance was measured at 734 nm. Ascorbic acid (vitamin C), SP, and SA were used as references. The following equation was used to calculate ABTS^+^ inhibition rate (%):

(6) $$ABTS^{+}\;inhibition\;rate\left(\%\right)=\frac{A_{ABTS}-A_{sample+ABTS}}{A_{ABTS}}100\;\left(\%\right)$$

where *A_ABTS_* is the absorbance of *ABTS^+^* and *A_sample+ABTS_* is the absorbance of *ABTS^+^* with the sample.

All measurements for all sample solutions at each concentration were performed in triplicate.

#### 3.6.3. Calculation of IC50

The IC50 (inhibitory concentration) value is the antioxidant concentration required to scavenge 50% of DPPH and ABTS free radicals. These values were calculated from the graph of radical scavenging activity against the different concentrations of tested samples. The concentration of the sample solution at IC50 (µg/mL) was determined by regression.

### 3.7. Data Analysis

Results are presented as the means ± S.D. (standard deviation) from three replicates of each experiment. All tests were considered significant at *p* < 0.05. Statistical analyses were performed using Rstudio 3.6.1 (Boston, MA, USA) open-source code. All figures were implemented by using the OriginPro 8.5 software (Northampton, MA, USA). The chemical structures of phenolic compounds were represented using the ChemDrawUltra 8.0 package (Cambridge Soft, MA, USA).

## 4. Conclusions

In conclusion, this study provides insights into the determination of phenolic compounds in an aqueous protein extraction/purification by-product (permeate) from rapeseed meal (RSM). We found that sinapine was the main phenolic compound in this permeate. The other compounds were sinapoyl glucose, 1,2-di-sinapoyl gentiobise, and sinapic acid. The acidic hydrolysis of this permeate allowed the efficient conversion of sinapine into sinapic acid. The XAD16 resin showed the highest adsorption capacity. Adsorption behaviors of total phenolic compounds, sinapine, and sinapic acid were also investigated. Our results revealed that the adsorption of phenolic compounds in both the hydrolyzed and non-hydrolyzed fractions followed the pseudo-second-order model and presented a very similar pattern of intra-particle diffusion. The Langmuir model was suitable to describe the adsorption process of phenolic compounds. Furthermore, the adsorption process was an exothermic, randomness, and physical adsorption process. Finally, we showed that the hydrolyzed fraction has the potential to become an interesting natural antioxidant, by comparison to vitamin C, that could find applications in food preservation or other domains.

## Figures and Tables

**Table 1:** Retention time (t_R_) and MS data of the peaks detected by HPLC-DAD and HPLC-MS in permeate and hydrolyzed permeate from rapeseed meal.

Peak	Compound Identity	t_R_ (Min)	*m*/*z*	Molecular Weight
1	Sinapine	18.94	310 [M − H]^+^	310
2	Sinapoyl glucose	23.31	385 [M − H]^−^	386
3	1,2-di-sinapoyl gentiobise	28.33	753 [M − H]^−^	754
4	Sinapic acid	31.68	223 [M − H]^−^	224

**Table 2:** Parameters of the calibration curves for pure sinapine and sinapic acid.

Phenolic Compounds	Regression Coefficient (R^2^)	LOD (µg/mL)	LOQ (µg/mL)
Sinapine	0.9998	0.25	0.60
Sinapic Acid	0.9984	0.25	0.60

**Table 3:** Kinetic parameters for sinapine and sinapic acid adsorption onto XAD16 resin in non-hydrolyzed and hydrolyzed permeates.

Kinetic Model	Equation	Parameter	Phenolic Compound	
			Sinapine	Sinapic Acid
Pseudo-first-order (PFO)	$\ln\left(q_{e}-q_{t}\right)=\ln\left(q_{e}\right)-k_{1}t$	*k*_1_ (1/min)	0.0627	0.0619
		*q_e,exp_* (mg/g)	13.532	3.861
		*q_e,cal_* (mg/g)	3.83	0.29
		R^2^	0.9381	0.8113
Pseudo-second-order (PSO)	$\frac{1}{q_{t}}=\frac{1}{k_{2}q_{e}^{2}}+\frac{t}{q_{e}}$	*k*_2_ (g/mg × min)	0.0733	0.2587
		*q_e,exp_* (mg/g)	13.532	3.861
		*q_e,cal_* (mg/g)	13.544	3.861
		R^2^	1	1
Intra-particle diffusion	$\;q_{t}=kt^{0.5}+C$	*k*_*i*,1_ (mg/g)/min^0.5^	1.0321	0.09
		*C*_1_ (mg/g)	8.9921	3.5118
		R^2^	0.9828	0.9639
		*k*_*i*,2_ (mg/g)/min^0.5^	0	0
		*C*_2_ (mg/g)	13.532	3.861

**Table 4:** Adsorption isotherm equation and parameters of sinapine, and sinapic acid adsorption onto XAD16 resin.

Isotherm Model	Parameter	Phenolic Compound	
		Sinapine	Sinapic Acid
Langmuir $q_{e}=\frac{Q_{max}K_{L}C_{e}}{1+K_{L}C_{e}}$	*Q_max_* (mgSAE/g)	35.93	23.96
	*K_L_* (L/mg)	124.29	0.076
	R^2^	0.997	0.9999
Freundlich $q_{e}=K_{F}C_{e}^{n}$	*K_F_* (mg/g)/(mg/L)^n^	0.1988	0.1562
	*n*	0.9987	0.9892
	R^2^	0.996	0.9978

**Table 5:** Thermodynamic parameters of sinapine and sinapic acid adsorption onto XAD16 at 25 °C, 35 °C, and 45 °C.

Phenolic Compound	Temperature (°C/K)	ΔS (kJ/molK)	ΔH (kJ/mol)	ΔG (kJ/mol)
Sinapine	25/298.15	−55.95	−2.56	−13.34
	35/308.15			−17.28
	45/318.15			−15.56
Sinapic Acid	25/298.15	−8.37	−2.72	−0.267
	35/308.15			−0.08
	45/318.15			−0.03

**Table 6:** IC50 values of compounds tested using the DPPH and ABTS assays.

Antioxidant	IC50/DPPH (µg/mL)	IC50/ABTS (µg/mL)
N fraction	10.05 ± 0.025a	22.70 ± 0.021a
H fraction	4.26 ± 0.012b	10.60 ± 0.029b
Sinapine Standard	7.58 ± 0.017c	17.98 ± 0.016c
Sinapic Acid Standard	2.16 ± 0.001d	5.51 ± 0.010d
Vitamin C	7.27 ± 0.019e	36.31 ± 0.021e

Different letters in the same column for the IC50/DPPH and IC50/ABTS indicate significant differences (*p* < 0.005) between individual sample treatments.

**Table 7:** Characteristics of the resins used in this study.

Resins	Material	Polarity	Specific Surface (m^2^/g)	Pore (Å)
XAD 4	SDVB*	Non-polar	725	50
XAD 7	Acrylate	Polar	450	90
XAD 16	SDVB	Non-polar	900	100
XAD 1180	SDVB	Non-polar	600	300
HP 20	SDVB	Non-polar	500	260

SDVB*: Styrene-divinyl benzene.

## Data Availability

Not applicable.

## Sample Availability

Samples of the compounds are not available from the authors.

## Abbreviations

HPLC	High-performance liquid chromatography
DAD	Diode-array detector
ESI	Electro-spray ionization
MS	Mass spectrometry
IT	Ion trap
SE-HPLC	Size exclusion high-performance liquid chromatography
UV	Ultraviolet
SAE	Sinapic acid equivalent
DPPH	1,1-diphenyl-2-picrylhydrazyl
ABTS	2,2′-azino-bis-(3-ethylbenzothiazoline-6-sulfonic acid)
IC50	Inhibitory concentration at 50% of scavenging rate
SP	Sinapine
SA	Sinapic acid
SG	Sinapoyl glucose
DV	Diavolume
UF	Ultra filtration
TPC	Total phenolic content
SDVB	Styrene-divinyl benzene
*q_e_*	Adsorption capacity (mg/g)
*C*_0_	Initial concentration of phenolic compound (mg/mL)
*C_e_*	Equilibrium concentration of phenolic compound (mg/mL)
*V_i_*	Volume of the initial sample solution (mL)
*W*	Weight of the tested dry resin (g)
*C_d_*	Concentration of phenolic compounds in desorption solution (mg/mL)
*V_d_*	Desorption solution volume (mL)
PFO	Pseudo-first-order
PSO	Pseudo-second-order
*q_t_*	Amount of adsorbate uptake per mass of adsorbent at any time t (min)
*k*_1_	Rate constant of the PFO equation (1/min);
*k*_2_	Rate constant of the PSO equation(g/mg×min)
*k_i_*	Rate constant of the intra-particle diffusion model (mg/g×min0.5)
*C*	Constant associated with the thickness of the boundary layer (mg/g)
*Q_max_*	Maximum saturated monolayer adsorption capacity of polyphenol (mg/g)
*K_L_*	Langmuir constant (L/mg)
*K_F_*	Freundlich constant (mg/g)/(mg/L)n
*n*	Freundlich intensity parameter (0 < n ≤1)
∆H	Enthalpy change (kJ/mol)
∆S	Entropy change (kJ/mol K)
∆G	Gibbs free energy change (kJ/mol K)
R	Ideal gas constant (8.314 J/mol K)
T	Temperature (K)
K_eq_	Equilibrium distribution coefficient of adsorption isotherm
S.D.	Standard deviation

## References

[1] Food and Agriculture Organization of the United Nations FAOSTAT. http://www.fao.org/faostat/en/#data/QC/visualize.

[2] Lomascolo A., Uzan-Boukhris E., Sigoillot J.-C., Fine F. (2012). Rapeseed and Sunflower Meal: A Review on Biotechnology Status and Challenges. Appl. Microbiol. Biotechnol..

[3] Naczk M., Amarowicz R., Sullivan A., Shahidi F. (1998). Current Research Developments on Polyphenolics of Rapeseed/Canola: A Review. Food Chem..

[4] Nićiforović N., Abramovič H. (2014). Sinapic Acid and Its Derivatives: Natural Sources and Bioactivity: Sinapic Acid and Its Derivatives. Compr. Rev. Food Sci. Food Saf..

[5] Kozlowska H., Zadernowski R., Sosulski F.W. (1983). Phenolic Acids in Oilseed Flours. Nahrung.

[6] Bell J.M. (1993). Factors Affecting the Nutritional Value of Canola Meal: A Review. Can. J. Anim. Sci..

[7] Thiyam U., Stöckmann H., Schwarz K. (2006). Antioxidant Activity of Rapeseed Phenolics and Their Interactions with Tocopherols during Lipid Oxidation. J. Am. Oil Chem. Soc..

[8] Khattab R., Eskin M., Aliani M., Thiyam U. (2010). Determination of Sinapic Acid Derivatives in Canola Extracts Using High-Performance Liquid Chromatography. J. Am. Oil Chem. Soc..

[9] Engels C., Schieber A., Gänzle M.G. (2012). Sinapic Acid Derivatives in Defatted Oriental Mustard (*Brassica Juncea* L.) Seed Meal Extracts Using UHPLC-DAD-ESI-MS n and Identification of Compounds with Antibacterial Activity. Eur. Food Res. Technol..

[10] Siger A., Czubinski J., Dwiecki K., Kachlicki P., Nogala-Kalucka M. (2013). Identification and Antioxidant Activity of Sinapic Acid Derivatives in Brassica Napus L. Seed Meal Extracts: Main Phenolic Compounds in Rapeseed. Eur. J. Lipid Sci. Technol..

[11] Wanasundara U.N., Shahidi F. (1994). Canola Extract as an Alternative Natural Antioxidant for Canola Oil. J. Am. Oil Chem. Soc..

[12] Thiyam U., Stöckmann H., Zum Felde T., Schwarz K. (2006). Antioxidative Effect of the Main Sinapic Acid Derivatives from Rapeseed and Mustard Oil By-Products. Eur. J. Lipid Sci. Technol..

[13] Krygier K., Sosulski F., Hogge L. (1982). Free, Esterified, and Insoluble-Bound Phenolic Acids. 2. Composition of Phenolic Acids in Rapeseed Flour and Hulls. J. Agric. Food Chem..

[14] Thiel A., Muffler K., Tippkötter N., Suck K., Sohling U., Hruschka S.M., Ulber R. (2015). A Novel Integrated Downstream Processing Approach to Recover Sinapic Acid, Phytic Acid and Proteins from Rapeseed Meal: A Novel Downstream Processing Approach for Rapeseed Meal. J. Chem. Technol. Biotechnol..

[15] Nadathur S., Wanasundara J.P., Scanlin L., Nadathur S., Wanasundara J.P.D., Scanlin L. (2016). Sustainable Protein Sources.

[16] Thiyam-Holländer U., Schwarz K. (2012). Rapeseed and canola phenolics: Antioxidant attributes and efficacy. Canola and Rapeseed.

[17] Downey R.K., Bell J.M., Shahidi F. (1990). New Developments in Canola Research. Canola and Rapeseed: Production.

[18] Quinn L., Gray S.G., Meaney S., Finn S., Kenny O., Hayes M. (2017). Sinapinic and Protocatechuic Acids Found in Rapeseed: Isolation, Characterisation and Potential Benefits for Human Health as Functional Food Ingredients. Ir. J. Agric. Food Res..

[19] Tzeng Y.-M., Diosady L.L., Rubin L.J. (1990). Production of Canola Protein Materials by Alkaline Extraction, Precipitation, and Membrane Processing. J. Food Sci..

[20] Defaix C., Aymes A., Albe Slabi S., Basselin M., Mathé C., Galet O., Kapel R. (2019). A New Size-Exclusion Chromatography Method for Fast Rapeseed Albumin and Globulin Quantification. Food Chem..

[21] Moreno-González M., Girish V., Keulen D., Wijngaard H., Lauteslager X., Ferreira G., Ottens M. (2020). Recovery of Sinapic Acid from Canola/Rapeseed Meal Extracts by Adsorption. Food Bioprod. Process..

[22] Weisz G.M., Schneider L., Schweiggert U., Kammerer D.R., Carle R. (2010). Sustainable Sunflower Processing—I. Development of a Process for the Adsorptive Decolorization of Sunflower [*Helianthus Annuus* L.] Protein Extracts. IFSE.

[23] Scordino M., Di Mauro A., Passerini A., Maccarone E. (2003). Adsorption of Flavonoids on Resins: Hesperidin. J. Agric. Food Chem..

[24] Sun P.-C., Liu Y., Yi Y.-T., Li H.-J., Fan P., Xia C.-H. (2015). Preliminary Enrichment and Separation of Chlorogenic Acid from Helianthus Tuberosus L. Leaves Extract by Macroporous Resins. Food Chem.

[25] Firdaous L., Fertin B., Khelissa O., Dhainaut M., Nedjar N., Chataigné G., Ouhoud L., Lutin F., Dhulster P. (2017). Adsorptive Removal of Polyphenols from an Alfalfa White Proteins Concentrate: Adsorbent Screening, Adsorption Kinetics and Equilibrium Study. Sep. Purif. Technol..

[26] Le T.T., Aymes A., Framboisier X., Ioannou I., Kapel R. (2020). Adsorption of Phenolic Compounds from an Aqueous By-Product of Sunflower Protein Extraction/Purification by Macroporous Resins. J. Chromatogr. A.

[27] Defaix C., Kapel R., Galet O. Protein Isolate and Process for the Production Thereof. https://patents.justia.com/inventor/claire-defaix.

[28] Albe-Slabi S., Defaix C., Beaubier S., Galet O., Kapel R. (2021). Selective Extraction of Napins: Process Optimization and Impact on Structural and Functional Properties. Food Hydrocoll..

[29] ICH I. Validation of Analytical Procedures: Text and Methodology. https://database.ich.org/sites/default/files/Q2%28R1%29%20Guideline.pdf.

[30] Yang Q., Zhao M., Lin L. (2016). Adsorption and Desorption Characteristics of Adlay Bran Free Phenolics on Macroporous Resins. Food Chem..

[31] Dong Y., Zhao M., Sun-Waterhouse D., Zhuang M., Chen H., Feng M., Lin L. (2015). Absorption and Desorption Behaviour of the Flavonoids from Glycyrrhiza Glabra L. Leaf on Macroporous Adsorption Resins. Food Chem..

[32] Xu Z., Zhang Q., Chen J., Wang L., Anderson G.K. (1999). Adsorption of Naphthalene Derivatives on Hypercrosslinked Polymeric Adsorbents. Chemosphere.

[33] Lagergren S. (1898). About the Theory of So-Called Adsorption of Soluble Substances. Kungliga Svenska Vetenskapsakad. Handlingarl..

[34] Ho Y.S., Mckay G. (1998). Kinetic Models for the Sorption of Dye from Aqueous Solution by Wood. Process Saf. Environ. Prot..

[35] Weber W.J., Morris J.C. (1963). Kinetics of Adsorption on Carbon from Solution. J. Sanit. Eng. Div..

[36] Annadurai G., Krishnan M.R.V. (1997). Adsorption of Acid Dye from Aqueous Solution by Chitin: Equilibrium studies. Indian J. Chem. Technol..

[37] Chen Y., Zhang W., Zhao T., Li F., Zhang M., Li J., Zou Y., Wang W., Cobbina S.J., Wu X. (2016). Adsorption Properties of Macroporous Adsorbent Resins for Separation of Anthocyanins from Mulberry. Food Chem..

[38] Liu B., Dong B., Yuan X., Kuang Q., Zhao Q., Yang M., Liu J., Zhao B. (2016). Enrichment and Separation of Chlorogenic Acid from the Extract of Eupatorium Adenophorum Spreng by Macroporous Resin. J. Chromatogr. B Biomed. Appl..

[39] Weisz G.M., Carle R., Kammerer D.R. (2013). Sustainable Sunflower Processing—II. Recovery of Phenolic Compounds as a by-Product of Sunflower Protein Extraction. Innov. Food Sci. Emerg. Technol..

[40] Thiel A., Tippkötter N., Suck K., Sohling U., Ruf F., Ulber R. (2013). New Zeolite Adsorbents for Downstream Processing of Polyphenols from Renewable Resources. Eng. Life Sci..

[41] Gao Z.P., Yu Z.F., Yue T.L., Quek S.Y. (2013). Adsorption Isotherm, Thermodynamics and Kinetics Studies of Polyphenols Separation from Kiwifruit Juice Using Adsorbent Resin. J. Food Eng..

[42] Gupta V.K. (1998). Equilibrium Uptake, Sorption Dynamics, Process Development, and Column Operations for the Removal of Copper and Nickel from Aqueous Solution and Wastewater Using Activated Slag, a Low-Cost Adsorbent. Ind. Eng. Chem. Res..

[43] Ding L., Deng H., Wu C., Han X. (2012). Affecting Factors, Equilibrium, Kinetics and Thermodynamics of Bromide Removal from Aqueous Solutions by MIEX Resin. Chem. Eng. J..

[44] Vuorela S., Meyer A.S., Heinonen M. (2003). Quantitative Analysis of the Main Phenolics in Rapeseed Meal and Oils Processed Differently Using Enzymatic Hydrolysis and HPLC. Eur. Food Res. Technol..

[45] Leyton A., Vergara-Salinas J.R., Pérez-Correa J.R., Lienqueo M.E. (2017). Purification of Phlorotannins from Macrocystis Pyrifera Using Macroporous Resins. Food Chem..

[46] Le T.T., Ropars A., Aymes A., Frippiat J.-P., Kapel R. (2021). Multicriteria Optimization of Phenolic Compounds Capture from a Sunflower Protein Isolate Production Process By-Product by Adsorption Column and Assessment of Their Antioxidant and Anti-Inflammatory Effects. Foods.

[47] Le T.T., Ropars A., Sundaresan A., Crucian B., Choukér A., Frippiat J.-P., Choukèr A. (2020). Pharmacological Countermeasures to Spaceflight-Induced Alterations of the Immune System. Stress Challenges and Immunity in Space.

[48] Yemm E.W., Willis A.J. (1954). The Estimation of Carbohydrates in Plant Extracts by Anthrone. Biochem. J..

[49] AOCS International (1995). Official Method 991.20. Nitrogen (Total) in Milk. Official Methods of Analysis.

[50] Wu C., Huang M., Lin Y., Ju H., Ching H. (2007). Antioxidant Properties of Cortex Fraxini and Its Simple Coumarins. Food Chem..

[51] Re R., Pellegrini N., Proteggente A., Pannala A., Yang M., Rice-Evans C. (1999). Antioxidant Activity Applying an Improved ABTS Radical Cation Decolorization Assay. Free Radic. Biol. Med..

